# Trends in dental care utilisation among the elderly using longitudinal data from 14 European countries: A multilevel analysis

**DOI:** 10.1371/journal.pone.0286192

**Published:** 2023-06-09

**Authors:** Nóra Kovács, Orsolya Liska, Enoabasi Omonigho Idara-Umoren, Nour Mahrouseh, Orsolya Varga

**Affiliations:** 1 Department of Public Health and Epidemiology, Faculty of Medicine, University of Debrecen, Debrecen, Hungary; 2 Faculty of Dentistry, Clinical Center, University of Debrecen, Debrecen, Hungary; 3 Faculty of Medicine, University of Debrecen, Debrecen, Hungary; 4 Office for Supported Research Groups, Eötvös Loránd Research Network, Budapest, Hungary; ISEG Lisbon School of Economics and Management, PORTUGAL

## Abstract

**Background:**

The use of dental care among older people is low compared to other forms of health care, with significant health consequences. However, the evidence on the extent to which countries’ welfare systems and socio-economic factors influence the uptake of dental care by older people is limited. This study aimed to describe trends of dental care utilisation, and to compare use of dental care with other types of healthcare services among the elderly, considering different socio-economic factors and welfare systems in European countries.

**Methods:**

Multilevel logistic regression analysis was performed using longitudinal data from four waves (between Wave 5 and 8) of the Survey of Health, Ageing and Retirement in Europe database, with a follow-up period of 7 years. The study sample included 20,803 respondents aged 50 years or older from 14 European countries.

**Results:**

The annual dental care attendance was the highest in Scandinavian countries (85.7%), however, improving trends of dental attendance was recognized in Southern and Bismarckian countries (p<0.001). The difference in use of dental care services between socio-economic groups was expanding over time regarding low- and high-income level and residential area. A more marked difference was observed between social groups in dental care utilisation compared to other forms of care. Income level and unemployed status had significant effect on forgoing dental care due to cost and unavailability.

**Conclusion:**

The observed differences between socioeconomic groups may highlight the health consequences of the different organization and financing of dental care. The elderly population could benefit from adopting policies aiming to reduce the financial barriers to dental care usage, especially in Southern and Eastern European countries.

## Introduction

In the high- and middle-income member states of the European Union (EU), oral diseases are still representing a major public health challenge: dental caries in permanent teeth, periodontal diseases, edentulousness, and other oral conditions accounted for 2.31 million Disability-Adjusted Life Years in 2019 [1, 2]. The more economically developed countries, generally, have the lowest rates of untreated tooth decay and severe periodontitis. Accordingly, within the EU, dental caries is a problem mostly for people from Eastern Europe and socio-economically disadvantaged groups covering elderly [3]. Oral diseases are not evenly distributed in society, over 50% of the European population have a form of periodontitis and it is increasing with age [3]. Natural ageing makes the elderly more susceptible to oral diseases, yet regular use of dental care is rare for several reasons including high costs of treatment, fear, availability and accessibility of services, or lack of perception of need [4–6]. This creates a population with a high prevalence of oral and dental diseases such as dental caries, periodontal disease and multiple tooth loss. The EU has increasingly ageing population [7], and a healthy ageing population can be achieved with better access to healthcare services including dental care [8].

Chronic oral diseases influence the quality of life due to pain, chewing, speaking and aesthetic functions of the teeth [9–11]. Moreover, oral health is an important part of the general health and the two can affect each other [12]. Poor oral health is associated with general health and a range of chronic conditions, including frailty [13]. Frailty, which is considered a significant public health concern related to the ageing population, is defined as a state of increased vulnerability to stressors leading to adverse health outcomes such as dependency, disability and cognitive impairment [14]. Frailty has also been shown to have a negative impact on the use of dental services and oral self-care among older people [15].

There are no consistent statistics on the frequency of dental screening for different age groups in different countries. In general, older adults need more frequent dental screening because of the increased risk of age-related oral changes. Although older people are less likely to visit the dentist in several countries [4, 16], there are some countries where a higher frequency of use of dental care can be observed among elderly compared with younger people [17]. The frequency of dental check-ups can vary greatly from one individual to another, and there are many factors that could affect the use of dental services, including the type of health care system, dental insurance coverage, and socio-economic status (SES) including household income and education level [5, 18]. People with lower SES fail to seek dental care due to the lack of financial resources [19], that may result in multiple tooth loss and thus impaired quality of life. Catastrophic oral health expenditure can be prevented if access to dental care is guaranteed. In the EU, expenditure on dental care is more constrained, due to limited service packages and higher level of cost sharing. On average, only around 30% of dental treatment costs are paid for by national schemes or compulsory insurance [1]. In only three EU countries (Croatia, Germany, and Slovakia) more than half of total expenditure due to dental care is covered, and there are countries (Greece and Spain) where the costs of dental care for adults without a specific entitlement are not paid at all [1]. However, voluntary health insurance can play an important financial protection role when dental care is not included in a comprehensive care package but this depends significantly on income, which is typically lower among older people [1].

To compare health outcomes or health inequalities between countries, using the concept of the welfare state may serve the purpose better than relying on the health system alone, as health is highly dependent on socio-economic factors that governments influence through a complex network of policies. Despite the heterogeneity of functional capacity in older age groups, ageing is often associated with increased support needs, which makes welfare state policies particularly relevant for this age group [20]. The welfare state can be categorised and examined in many ways [21]. Building on the Esping-Andersen’s typology [22], Ferrera [23] introduced a modified typology (including Scandinavian, Anglo-Saxon, Bismarckian, and Southern welfare states) that focuses on differences in the way social benefits are provided rather than focusing on the quantity of welfare given [24]. An important step forward in the research on welfare regimes was the inclusion of a fifth type of regime by Eikemo et al. [25], the Eastern European regime which is characterised by a series of economic instability and social reforms [26]. A number of previous studies have investigated the link between welfare state regimes and oral health inequalities in a wide range of European countries, using the modified Ferrera typology [27–30].

Understanding the extent to which the uptake of dental care lags behind other medical care, how socio-economic factors determine the use of dental care, and the extent to which welfare systems can compensate for the uptake gap is important to enable an appropriate policy response. Thus, our aim is to describe the dental care utilisation, and to compare utilisation of dental care services with other types of health care services in European countries in populations over 50 years old.

## Materials and methods

### Data source

This study was a secondary data analysis of the Survey of Health, Ageing and Retirement in Europe (SHARE) survey, which is a cross-national panel study collecting data on health, social, and socioeconomic characteristics from individuals aged 50 years and older, as well as their partners [31]. Data are collected in 27 European countries and Israel. Four waves of the SHARE database were used for this longitudinal study, with a follow-up period of 7 years. The study is based on data from Waves 5 (2013), 6 (2015), 7 (2017), and 8 (2019/2020) of the SHARE survey [32–35]. All waves contain detailed retrospective data of respondents aged 50 years or older. Detailed information with regard to the SHARE data survey is indicated elsewhere [31].

SHARE has been continuously reviewed and approved by the Ethics Council of the Max Planck Society. The country implementations of the survey were reviewed and approved by the relevant ethics committees or institutional review boards in each country. All participants provided written informed consent and all data provided by SHARE are anonymized.

### Study samples

The longitudinal sample included 20,803 respondents aged 50 years or older who provided data on use of healthcare (medical doctor visits, dental care, hospitalization) and for covariates in all four waves of SHARE. Participants with other citizenship than the investigated countries were excluded from our study. Since the study is based on longitudinal data at four cross-sectional time points, we used data from those 14 European countries where data from all four waves were available: Denmark, Sweden, Austria, Belgium, France, Germany, Luxembourg, the Netherlands, Switzerland, Italy, Spain, Czech Republic, Estonia, and Slovenia.

### Measurements

#### Demographic, socioeconomic and health variables

The demographic variables in the analysis included gender, age (respondents aged 50 years and older at the time of interview). Four individual-level variables were used as indicators of SES including educational attainment, income level, employment status and residential area. Educational attainment was coded to three categories using International Standard Classification of Education (ISCED): none/primary (ISCED scores 0 and 1), secondary (ISCED scores 2–4), and tertiary (ISCED scores 5 and 6) [36]. We used the self-reported household (after-tax) income and created four categories for the average monthly household income for each country, as follows: low income (0-25^th^ percentile), middle income (25^th^-50^th^ percentile), upper middle income (50^th^-75^th^ percentile), and high income (75^th^ percentile or higher). Income of non-respondents was calculated based on the provided imputations for each wave of SHARE. Employment status was categorized as employed, unemployed, permanently sick or disabled, retired and other (including homemakers), and the residential area was classified as urban or rural. Other variables regarding health status were also included in the models, as activity limitation (based on the Global Activity Limitation Index [37], number of chronic diseases (whether the respondent reported being diagnosed with chronic disease: 0, 1 and 2 or more), self-perceived health (excellent, very good, good, fair, and poor), and use of any medication.

To investigate how different European welfare state types affect the healthcare attendance, the 14 countries were categorized into the following four welfare state regimes: Scandinavian (Denmark, Sweden), Bismarckian (Austria, Belgium, France, Germany, Luxembourg, the Netherlands, and Switzerland), Southern (Italy, and Spain), and Eastern (Czech Republic, Estonia, and Slovenia) [22, 38].

#### Outcome variables

The use of dental care, medical care and hospitalization was explored in this study. Participants were asked the questions “During the last twelve months, have you seen a dentist or a dental hygienist?”, “During the last 12 months, about how many times in total have you seen or talked to a medical doctor or qualified/registered nurse about your health?”, “During the last twelve months, have you been in a hospital overnight?”. Regarding the medical doctor/nurse visits, a binary variable was created based on the numeric answers, which were classified as “0” and “At least 1”.

Data from Wave 8 include information about forgoing dental care and its reasons (cost and unavailability/inaccessibility). The participants in wave 8 were asked about “During the last twelve months, which of the following types of care did you forgo because of the costs you would have to pay, if any?” and “During the last twelve months, which of the following types of care did you forgo because they were not available or not easily accessible, if any?” followed by a list of types of care including dental care.

#### Data analysis

Descriptive statistics were performed to describe the baseline study sample. Study participants were aggregated into groups based on attendance, and percentages of respondents for those attended and not attended healthcare were calculated across explanatory variables. The attendance rate was calculated for SES indicators and urbanization by baseline characteristics of participants to investigate the change over time.

The Slope Index of Inequality (SII) and the Relative Index of Inequality (RII) were used to measure the absolute and relative inequalities in use of dental care and healthcare services according to SES indicators with hierarchical order (education and income level) [39]. The ridit score for estimating SII and RII was calculated [40]. Rate ratio for Poisson regression (considered as RII) and beta coefficient for linear regression (considered as SII) with 95% CI were reported. All models were adjusted for age, gender, place of residence, employment status, number of chronic diseases, activity limitation, medication use, self-reported health status and country. Educational and income level at each wave were transformed into a ridit score (scaled between 0 and 1) corresponding to the midpoint of the cumulative proportion of the population of each SES category.

The SII can be interpreted as the absolute difference in the healthcare attendance experience between the highest and lowest level SES. On the other hand, RII is the ratio of reporting healthcare attendance between the higher and lower levels of SES groups. A SII value higher than 0 and a RII value higher than 1 indicate that the use of healthcare services is more common among those with higher SES.

Chi-square test for trend analysis was performed for proportions of healthcare attendance during study period across each welfare state regime and SES indicators.

We used multilevel models, where the repeated individual observations (level 1) are nested within countries (level 2). We constructed a mixed-effect multilevel logistic regression model (as the outcome measures were binary) investigating the associations between use of medical, dental care and hospitalization and demographic, SES, urbanization and health variables.

In addition, we constructed two models separately for reasons of forgoing dental care based on data from Wave 8. First, we tested whether the odds of reporting forgone dental care varied across SES markers in Model 1, adjusted for individual level variables (age, gender, self-perceived health, number of chronic diseases, medication use, and activity limitation). In Model 2 a country-level variable (welfare state regime) was added to the previous model.

Since oral health status might be associated with use of dental care services, we conducted the following sensitivity analyses: First, a multiple imputation was used regarding missing data in wave 7, with pain in teeth/mouth variable (N = 14,389) being imputed and added to the model as potential predictor. Secondly, the analysis was performed only for the baseline year (in wave 5), and pain in teeth/mouth and number of teeth (available only in wave 5) variables were added to the model.

The model fitness was assessed using Akaike Information Criterion (AIC), where the smaller AIC value is indicating a better model fit. The intraclass correlation (ICC) was calculated to measure the degree of clustering of individuals within the same country. Odds ratios (OR) and the corresponding 95% confidence intervals (CI) were presented. P-values smaller than 0.05 were considered statistically significant. All analyses were performed in Stata (version 13, Stata Corp., USA).

## Results

The study sample consisted of 20,803 participants aged 50 years and older from 14 European countries, who were available during the study period (2013–2020). The mean age of participants was 65.33 (SD 8.57) at baseline (in 2013), and 57.5% of the sample were women. Overall, 16.4% of participants had none or low level of education, two-third (65.5%) of them were living in urban areas, and more than half of the respondents (57.5%) were retired. Almost half (47.8%) of them had under the median household annual net income. Majority of the study participants (44.3%) came from Bismarckian welfare state. 63.4% of the respondents visited the dentist, 89.8% seen a medical doctor and 13% of them were admitted to hospital in the preceding 12 months (Table 1).

**Table 1:** Distribution of study sample by dental care attendance, medical care attendance and hospitalization, by demographic, socioeconomic and health variables and welfare state regimes, at baseline (in 2013).

	All participants	Participants who attended dental care in the last 12 months	Participants who attended medical care in the last 12 months	Participants who were hospitalized in the last 12 months
	N = 20803	N = 13179 (63.4%)	N = 18682 (89.8%)	N = 2708 (13%)
**Age, years (mean, SD)**	65.33 (8.57)	64.57 (8.18)	65.58 (8.61)	66.75 (8.79)
**Age, years**				
50–54	2242 (10.8%)	1537 (11.7%)	1935 (10.4%)	248 (9.2%)
55–59	3665 (17.6%)	2465 (18.7%)	3204 (17.2%)	392 (14.5%)
60–64	4302 (20.7%)	2840 (21.5%)	3791 (20.3%)	493 (18.2%)
65–69	4069 (19.6%)	2693 (20.4%)	3648 (19.5%)	535 (19.8%)
70–74	3217 (15.5%)	1970 (14.9%)	2990 (16%)	501 (18.5%)
>75	3308 (15.9%)	1674 (12.7%)	3114 (16.7%)	539 (19.9%)
**Gender**				
Female	11953 (57.5%)	7644 (58%)	10892 (58.3%)	1582 (58.4%)
Male	8850 (42.5%)	5535 (42%)	7790 (41.7%)	1126 (41.6%)
**Education**				
None/primary	3402 (16.4%)	1284 (9.7%)	3115 (16.7%)	464 (17.1%)
secondary	12016 (57.8%)	7721 (58.6%)	10761 (57.6%)	1605 (59.3%)
tertiary	5385 (25.9%)	4174 (31.7%)	4806 (25.7%)	639 (23.6%)
**Household income**				
Low income (0-25^th^ percentile)	4970 (23.9%)	2744 (20.8%)	4482 (24%)	761 (28.1%)
Middle income (25^th^-50^th^ percentile)	4980 (23.9%)	3049 (23.1%)	4533 (24.3%)	663 (24.5%)
Upper middle income (50^th^-75^th^ percentile)	5390 (25.9%)	3651 (27.7%)	4878 (26.1%)	673 (24.9%)
High income (75^th^ percentile and higher)	5463 (26.3%)	3735 (28.3%)	4789 (25.6%)	611 (22.6%)
**Residential area**				
Rural	7171 (34.5%)	4359 (33.1%)	6402 (34.3%)	967 (35.7%)
Urban	13632 (65.5%)	8820 (66.9%)	12280 (65.7%)	1741 (64.3%)
**Employment status**				
Employed	6140 (29.5%)	4402 (33.4%)	5227 (28%)	539 (19.9%)
Other/homemaker	1611 (7.7%)	796 (6%)	1464 (7.8%)	206 (7.6%)
Permanently sick or disabled	555 (2.7%)	303 (2.3%)	535 (2.9%)	151 (5.6%)
Retired	11962 (57.5%)	7406 (56.2%)	11003 (58.9%)	1755 (64.8%)
Unemployed	535 (2.6%)	272 (2.1%)	453 (2.4%)	57 (2.1%)
**Self-perceived health**				
Excellent	2013 (9.7%)	1584 (12%)	1537 (8.2%)	89 (3.3%)
Very good	4179 (20.1%)	3022 (22.9%)	3566 (19.1%)	294 (10.9%)
Good	8131 (39.1%)	5205 (39.5%)	7386 (39.5%)	890 (32.9%)
Fair	5137 (24.7%)	2775 (21.1%)	4885 (26.1%)	1007 (37.2%)
Poor	1343 (6.5%)	593 (4.5%)	1308 (7%)	428 (15.8%)
**Number of chronic diseases**				
0	4936 (23.7%)	3223 (24.5%)	3724 (19.9%)	243 (9%)
1	6396 (30.7%)	4115 (31.2%)	5832 (31.2%)	676 (25%)
2 or more	9471 (45.5%)	5841 (44.3%)	9126 (48.8%)	1789 (66.1%)
**Medication use**				
No	5548 (26.7%)	3728 (28.3%)	4131 (22.1%)	315 (11.6%)
Yes	15255 (73.3%)	9451 (71.7%)	14551 (77.9%)	2393 (88.4%)
**Activity limitations**				
Limited	10477 (50.4%)	6276 (47.6%)	9781 (52.4%)	1792 (66.2%)
Not limited	10326 (49.6%)	6903 (52.4%)	8901 (47.6%)	916 (33.8%)
**Welfare state regime**				
Scandinavian	3492 (16.8%)	3011 (22.8%)	2912 (15.6%)	357 (13.2%)
Bismarckian	9206 (44.3%)	6592 (50%)	8497 (45.5%)	1353 (50%)
Southern	2923 (14.1%)	858 (6.5%)	2618 (14%)	283 (10.5%)
Eastern European	5182 (24.9%)	2718 (20.6%)	4655 (24.9%)	715 (26.4%)

Overall, the highest percentage of participants with dental attendance within one year was found for the Scandinavian welfare system (85.7% in 2019/2020), followed by the Bismarckian (74.8%), Eastern European (53%), and Southern countries (38%). Regarding medical care attendance and hospitalization, lower variability was observed across welfare states (Table 2).

**Table 2:** Trend analysis for proportion of respondents who reported dental care, medical care, and hospital attendance during the preceding 12 months of interviews by welfare state regime.

	Wave 5 (2013) N (%)	Wave 6 (2015) N (%)	Wave 7 (2017) N (%)	Wave 8 (2019/2020) N (%)	p-value^a^
**Dental care**					
Scandinavian	3011 (86.2%)	3014 (86.3%)	3051 (87.4%)	2992 (85.7%)	0.825
Bismarckian	6592 (71.6%)	6633 (72.1%)	6910 (75.1%)	6887 (74.8%)	**<0.001**
Southern	858 (29.4%)	883 (30.2%)	968 (33.1%)	1111 (38%)	**<0.001**
Eastern European	2718 (52.5%)	2775 (53.6%)	2837 (54.7%)	2747 (53%)	0.354
**Medical care**					
Scandinavian	2912 (83.4%)	3001 (85.9%)	3013 (86.3%)	3084 (88.3%)	**<0.001**
Bismarckian	8497 (92.3%)	8545 (92.8%)	8586 (93.3%)	8665 (94.1%)	**<0.001**
Southern	2618 (89.6%)	2663 (91.1%)	2670 (91.3%)	2737 (93.6%)	**<0.001**
Eastern European	4655 (89.8%)	4767 (92%)	4768 (92%)	4845 (93.5%)	**<0.001**
**Hospitalisation**					
Scandinavian	357 (10.2%)	406 (11.6%)	421 (12.1%)	486 (13.9%)	**<0.001**
Bismarckian	1353 (14.7%)	1445 (15.7%)	1540 (16.7%)	1755 (19.1%)	**<0.001**
Southern	283 (9.7%)	298 (10.2%)	346 (11.8%)	368 (12.6%)	**<0.001**
Eastern European	715 (13.8%)	756 (14.6%)	783 (15.1%)	851 (16.4%)	**<0.001**

^a^ p-values were calculated using Chi-square test for trend

Chi-square test for trend analysis showed that use of healthcare services among participants increased in most countries. The highest improving trend in dental care attendance was observed in Southern (p<0.001) countries, followed by Bismarckian (p<0.001) countries, while the rate of attenders did not change significantly in Scandinavian and Eastern European countries between 2013 and 2020 (Table 2).

Social inequalities were observed more likely in dental care attendance compared with the other two forms of care (see S1–S3 Figs). Educational level showed the highest variability among patients regarding dental care attendance, people with lowest level showed a higher increase in attendance over time. It appears (especially in terms of income level and urbanization) that while the gap between SES groups was constant or has been declined over time in medical care and hospitalization, it was expanding in dental care. The gap in attendance rate between participants with low- and high-income level has increased from 13.3% to 17.5%, while from 3.5% to 4.6% among participants living in rural versus urban region (S1-S3 Figs).

After adjusting all covariates, absolute and relative income inequalities in use of dental care increased over the study period (p<0.001). Relative income inequalities increased from 1.14 (1.1–1.19) in wave 5 to 1.27 (1.22–1.32) in wave 8. For SII, the similar significantly increasing trend was observed, while relative educational inequalities slightly decreased (p = 0.049). However, both educational and income inequalities were higher for dental care attendance compared to medical care and hospitalization (Table 3), even after adjusting for the oral health related variables in the sensitivity analysis (S1 Table). According to the stratified estimates by welfare state regimes, the extent of educational and income inequalities in use of dental care varied more across welfare states than for other forms of care. A significant increasing trend was observed in relative and absolute income inequalities in Bismarckian and Southern countries, and not significant increase in Scandinavian countries (S2 Table).

**Table 3:** Relative (RII) and absolute (SII) inequalities related to dental care, healthcare attendance and hospitalization.

	RII (95% CI)				
	wave5	wave6	wave7	wave8	p-value#
**Education**					
Dental care	1.83 (1.75–1.9)**	1.8 (1.73–1.88)**	1.83 (1.76–1.9)**	1.72 (1.66–1.79)**	0.049
Medical care	1.04 (1.02–1.06)**	1.02 (1–1.04)*	1.03 (1.02–1.05)**	1.04 (1.02–1.05)**	0.234
Hospitalization	1.39 (1.2–1.6)**	1.2 (1.05–1.37)*	1.04 (0.92–1.19)	1.18 (1.05–1.33)*	0.064
**Income**					
Dental care	1.14 (1.1–1.19)**	1.19 (1.15–1.24)**	1.27 (1.22–1.31)**	1.27 (1.22–1.32)**	<0.001
Medical care	1.04 (1.02–1.06)**	1.04 (1.02–1.06)**	1.04 (1.02–1.06)**	1.04 (1.03–1.05)**	0.256
Hospitalization	1.08 (0.95–1.23)	1.1 (0.97–1.25)	0.97 (0.86–1.1)	0.95 (0.85–1.06)	0.039
	**SII (95% CI)**				
**Education**					
Dental care	0.407 (0.377–0.437)**	0.377 (0.347–0.408)**	0.394 (0.365–0.424)**	0.359 (0.328–0.39)**	0.178
Medical care	0.034 (0.014–0.054)*	0.014 (-0.005–0.033)	0.038 (0.019–0.058)**	0.041 (0.022–0.061)**	0.197
Hospitalization	0.014 (-0.006–0.034)	0.013 (-0.008–0.034)	-0.008 (-0.03–0.013)	-0.005 (-0.027–0.018)	0.033
**Income**					
Dental care	0.121 (0.094–0.148)**	0.145 (0.118–0.173)**	0.186 (0.159–0.213)**	0.192 (0.164–0.22)**	<0.001
Medical care	0.035 (0.018–0.052)**	0.041 (0.025–0.058)**	0.044 (0.027–0.061)**	0.042 (0.025–0.059)**	0.276
Hospitalization	-0.003 (-0.021–0.015)	0.009 (-0.009–0.028)	-0.008 (-0.028–0.011)	-0.015 (-0.036–0.005)	0.105

RII, Relative Index of Inequality, SII Slope Index of Inequality

All models were adjusted for age, gender, self-perceived health, number of chronic diseases, medication use, activity limitation and country.

*p<0.05

**p<0.001

#p-value for trend

According to the multilevel analysis, although the 55–59 and 60-64-years age groups showed significantly higher attendance rates, a continuous decline in dental care attendance was observed from 65 years onwards with significant association for those 75 years and older. Those with higher educational attainment and living in urban areas were more likely to attend both dentist and medical doctor within one year. The increasing level of average household income showed significant association with higher probabilities of dental and medical care attendance. Unemployed status was found to be significantly lowering the likelihood of having dental and medical check-up compared with employed participants. A higher number of comorbidities was significantly associated with the use of any type of service. Those with poorer self-reported health status used significantly less dental care, in contrast, the opposite trend was observed for medical care and hospitalization (Table 4).

**Table 4:** Multilevel logistic regression model for determinants of dental care attendance, medical care attendance and hospitalization.

	Dental care attendance	Medical care attendance	Hospitalization
	OR (95% CI)	OR (95% CI)	OR (95% CI)
**Age, years** (Reference: 50–54)			
55–59	1.14 (1.04–1.25)*	0.98 (0.87–1.11)	0.95 (0.84–1.09)
60–64	1.13 (1.03–1.24)*	0.98 (0.87–1.11)	0.93 (0.82–1.06)
65–69	1.06 (0.96–1.17)	1.06 (0.92–1.22)	0.94 (0.82–1.08)
70–74	0.99 (0.89–1.1)	1.19 (1.03–1.39)*	1.02 (0.88–1.17)
>75	0.69 (0.62–0.77)**	1.19 (1.02–1.39)*	1.12 (0.98–1.29)
**Gender** (Reference: Female)			
Male	0.71 (0.68–0.73)**	0.77 (0.73–0.81)**	1.19 (1.15–1.25)**
**Education** (Reference: None/primary)			
Secondary	1.64 (1.56–1.72)**	1.19 (1.09–1.3)**	1 (0.93–1.06)
Tertiary	2.48 (2.34–2.63)**	1.43 (1.29–1.58)**	1.04 (0.97–1.13)
**Household income** (Reference: Low income)			
Middle income	1.32 (1.26–1.38)**	1.39 (1.29–1.51)**	1.02 (0.96–1.08)
Upper middle income	1.58 (1.51–1.66)**	1.44 (1.33–1.56)**	1 (0.94–1.06)
High income	1.79 (1.71–1.89)**	1.5 (1.38–1.62)**	1.06 (0.99–1.12)
**Residential area** Reference: Rural)			
Urban	1.16 (1.12–1.2)**	1.16 (1.1–1.22)**	1.03 (0.98–1.07)
**Employment status** (Reference: Employed)			
Other/homemaker	0.84 (0.77–0.91)**	0.91 (0.81–1.03)	1.13 (1.01–1.25)*
Permanently sick	0.82 (0.73–0.92)*	1.48 (1.12–1.94)*	1.48 (1.31–1.68)**
Retired	0.97 (0.91–1.03)	1.1 (1.01–1.2)*	1.26 (1.16–1.37)**
Unemployed	0.7 (0.62–0.79)**	0.82 (0.69–0.97)*	0.99 (0.83–1.18)
**Number of chronic diseases** (Reference: 0)			
1	1.09 (1.03–1.15)*	1.58 (1.48–1.69)**	1.23 (1.13–1.34)**
2 or more	1.17 (1.11–1.24)**	3.01 (2.75–3.29)**	1.59 (1.47–1.73)**
**Activity limitations** (Reference: No)	0.97 (0.94–1.01)	1.32 (1.24–1.41)**	1.66 (1.58–1.74)**
**Medication use** (Reference: No)	1.11 (1.06–1.17)**	3.28 (3.07–3.51)**	1.47 (1.36–1.58)**
**Self-perceived health** (Reference: Excellent)			
Very good	0.99 (0.91–1.07)	1.3 (1.19–1.4)**	1.13 (1–1.28)
Good	0.86 (0.79–0.92)**	1.48 (1.37–1.61)**	1.56 (1.39–1.76)**
Fair	0.72 (0.67–0.78)**	1.68 (1.51–1.88)**	2.43 (2.15–2.75)**
Poor	0.55 (0.5–0.61)**	2.02 (1.67–2.44)**	4.21 (3.67–4.81)**
**Wave** (Reference: Wave 5)			
Wave 6	1.06 (1.01–1.11)*	1.16 (1.08–1.24)**	1.07 (1–1.13)*
Wave 7	1.24 (1.19–1.3)**	1.04 (0.96–1.12)	1.04 (0.98–1.1)
Wave 8	1.28 (1.22–1.34)**	1.15 (1.06–1.24)*	1.12 (1.05–1.18)**
**Welfare system** (Reference: Scandinavian)			
Bismarckian	0.49 (0.24–1.01)	2.29 (1.28–4.08)*	1.25 (0.87–1.8)
Southern	0.1 (0.04–0.25)**	1.48 (0.72–3.04)	0.67 (0.42–1.05)
Eastern European	0.18 (0.08–0.4)**	1.3 (0.67–2.5)	0.86 (0.57–1.29)
Intercept	2.74 (1.44–5.22)*	0.66 (0.39–1.13)	0.02 (0.02–0.03)**
ICC (%)	5.95%	3.89%	1.55%
AIC	88853	40542.3	63187.95

Total observations = 83,212, N participants = 20,803, N groups = 14

OR, Odds Ratio, CI, Confidence interval, AIC, Akaike Information Criterion, ICC, Intraclass Correlation coefficient

*p<0.05

**p<0.001

For the welfare state regime in the analyses, respondents from Southern (OR = 0.1 (0.04–0.25)), and Eastern European (OR = 0.18 (0.08–0.4)) countries were significantly less likely to having regular dental care visits, which was not observed for other types of healthcare services. A rate of annual dental care visits generally increased significantly over time among participants (2019/2020 vs. 2013: OR = 1.28 (1.22–1.34)), while a significant increase was also observed in rates of medical care visit (2019/2020 vs. 2017: OR = 1.15 (1.06–1.24)) and hospitalization (2019/2020 vs. 2017: OR = 1.12 (1.05–1.18)) from wave 7 to 8 (Table 4).

The analyses including oral health status variables (presence of pain in teeth/mouth and number of teeth), performed as sensitivity analyses, were consistent with the main results (S3 Table).

Relatively small number of participants reported any reason, including forgone dental care due to cost (N = 635, 3.06%) and due to unavailability/inaccessibility (N = 233, 1.12%). In multilevel models the income level and unemployment status showed significant association with forgoing dental care due to cost and unavailability. After adding welfare state regime as second level variable to the analysis (Model 2), the country level variance reduced (ICC = 7.81% and 7.05%). The Eastern European (OR = 4.00 (1.36–11.72)) and Southern (OR = 3.71 (1.14–12.04)) welfare states were significantly associated with forgone dental care due to unavailability, regardless of individual factors (Tables 5 and S4).

**Table 5:** Multilevel logistic regression analysis for forgoing dental care due to cost and unavailability in wave 8.

	Forgoing dental care due to cost		Forgoing dental care due to unavailability	
	Model 1^a^	Model 2^b^	Model 1^a^	Model 2^b^
	OR (95% CI)	OR (95% CI)	OR (95% CI)	OR (95% CI)
**Education** (Reference: None/primary)				
Secondary	0.96 (0.76–1.23)	0.97 (0.76–1.24)	0.95 (0.65–1.39)	0.95 (0.65–1.39)
Tertiary	1.02 (0.76–1.37)	1.03 (0.77–1.39)	0.91 (0.56–1.49)	0.93 (0.57–1.51)
**Household income** (Reference: Low income)				
Middle income	0.64 (0.52–0.8)**	0.64 (0.52–0.8)**	0.65 (0.46–0.91)**	0.65 (0.46–0.91)*
Upper middle income	0.53 (0.42–0.67)**	0.53 (0.42–0.67)**	0.42 (0.28–0.62)*	0.41 (0.28–0.62)*
High income	0.35 (0.27–0.45)**	0.35 (0.27–0.45)**	0.46 (0.31–0.68)**	0.46 (0.31–0.67)**
**Residential area** (Reference: Rural)				
Urban	1.17 (0.98–1.39)	1.16 (0.97–1.38)	1.2 (0.9–1.59)	1.19 (0.89–1.58)
**Employment status** (Reference: Employed)				
Other/Homemaker	1.75 (1.18–2.59)*	1.73 (1.17–2.57)*	1.33 (0.67–2.64)	1.31 (0.66–2.6)
Permanently sick	1.69 (1.08–2.65)*	1.69 (1.08–2.65)*	0.93 (0.39–2.23)	0.92 (0.38–2.22)
Retired	1.29 (0.94–1.77)	1.29 (0.94–1.77)	1.3 (0.77–2.19)	1.31 (0.78–2.22)
Unemployed	2.36 (1.38–4.03)*	2.34 (1.37–4)*	2.42 (1.05–5.6)*	2.36 (1.02–5.47)*
**Welfare system** (Reference: Scandinavian)				
Bismarckian		0.7 (0.29–1.68)		1.29 (0.47–3.53)
Southern		1.54 (0.52–4.56)		3.71 (1.14–12.04)*
Eastern European countries		1.34 (0.5–3.61)		4.00 (1.36–11.72)*
Intercept	0.05 (0.03–0.1)**	0.05 (0.02–0.14)**	0.01 (0–0.03)**	0 (0–0.02)**
ICC (%)	10.77%	7.81%	15.39%	7.05%
AIC	5333.046	5334.87	2408.099	2404.818

^a^ Model 1 were adjusted for age, gender, self-perceived health, number of chronic diseases, medication use, and activity limitation

^b^ Model 2 were adjusted for the same variables as Model 1 and welfare-state regime was added to the model. Total participants = 20,773, N groups = 14.

*p<0.05

**p<0.001

OR, Odds Ratio, CI, Confidence interval, AIC, Akaike Information Criterion, ICC, Intraclass Correlation coefficient.

## Discussion

This study compared the use of dental care with the use of medical services among the elderly by SES and welfare systems. While medical care attendance and hospitalization are increasing with age due to the general health condition, mobility and disability, the dental-care non-attendance increases from the age of 65 and is highest over 75 years. People in older age groups have limited access to dental care services for a number of reasons, including lack of dentists and long waiting time, but other common barriers are related to the cost of dental treatment, dental anxiety and fear, as Borreani and colleagues found [4]. Furthermore, the decrease in participation in regular screening can be explained by a decrease in demand for the service [41]. The perceived need for dental check-ups among people who use dental prostheses is decreasing, especially among older age groups [4]. In addition, frailty and functional disability in older ages may also have an impact on oral hygiene routines and regular dental visits [13]. Frailty in combination with a lack of belief that a dentist could improve their oral health can further contribute to lower use of dental care services [15].

We identified significant differences in attendance in dental care among the elderly between welfare state systems. The lowest level of annual non-attendance was found in Southern countries, followed by the Eastern European and the Bismarckian welfare-state regimes, while the highest attendance was observed in Scandinavian countries. A rate of annual dental care visits increased significantly over time in Bismarckian and Southern welfare state regimes, while respondents from Southern and Eastern European countries were significantly less likely to have regular dental care visits after adjusting for demographic, socio-economic and health-related factors. Our findings also showed a significant increase in income inequalities in Bismarckian and Southern welfare state regimes. The differences in dental care visits may partially be attributed to behavioral and cultural differences in how the need for access to dental care is embedded in health thinking [42]. Our findings suggest that the Scandinavian welfare state regime is associated with higher rate of dental care attendance, which is in line with the literature that suggests that the oral health of the Nordic population is better due to the more redistributive and universal welfare system [27], including better care for the elderly, as municipalities (partly) cover their dental costs, as in Denmark and Norway [43]. Sweden also provides special dental care subsidies for certain population groups [19]. Furthermore, unequal access to oral health services could be a reason for the development of oral health diseases for socially disadvantaged groups. Studies from Sweden showed that the lack of access to dental care services explained about 60% of the socioeconomic differences in poor oral health [44]. However, access to care is not the only issue, results of past studies showed that coverage of dental care costs may help in access and the usage of dental care services, but it is still scarce in socioeconomic disadvantaged groups [44].

We may speculate that the benefits and services provided by the Scandinavian and Bismarckian welfare regimes mitigate better barriers to doctor’s visit by those living at lower socioeconomic levels. Regional availability of healthcare services and providers and perception about oral health are further determinants of unequal utilisation of dental care services [45]. A recent study reports that the health system-level factor, referring to regional availability of services, and the perception of regular dental treatment as “not necessary” were more often reported as the reasons of dental non-attendance in the Southern, Eastern, and Bismarckian welfare-state regimes than in Scandinavian countries [30].

Health-related behaviors can also explain oral health inequalities across European welfare state regimes, particularly in Scandinavian and Eastern countries [46]. Previous research found an independent cultural effect in the perceived oral impact on quality of life and the need of treatment among elderlies in Britain and Greece [47]. In addition, others have found that lower SES is a risk factor for poor oral health knowledge and inadequate oral hygiene habits [48], while higher health literacy is associated with better oral health behaviours [49].

The disparities in the use of dental care between social groups were greater than for other health services. Elderlies with higher incomes had significantly higher use of dental care over time than those with lower incomes. More general poverty may play an important role when families/individuals save to use other health services. Lower-income families who reported financial hardship are less likely to be able to afford health services and more likely to postpone or cancel medical visits [50, 51]. Out-of-pocket costs can result in a higher level of unmet needs for dental care due to financial reasons compared to other types of care [52]. However, public coverage of dental care does not necessarily mean that people in any country have unlimited access to dental services. There is some evidence that cost barriers for older adults occur regardless of the cost coverage of dental care services [53]. In most countries, the normative coverage is limited to standard materials; materials that provide higher quality dental care and thus better health outcomes have to be paid for out-of-pocket by the patient. Financial protection measures exist in some countries for specific population groups, such as low-income or other vulnerable groups (the elderly). However, even mitigating measures such as the high-cost protection scheme in Sweden do not necessarily fully alleviate out-of-pocket burdens [54]. At European level, there are currently no detailed common guidelines for the treatment and management of patients with oral health problems, which makes it difficult to compare the coverage and accessibility of oral health services [19].

Similarly to our results, others found different patterns of dental attendance between urban and rural residents [55–57], which may suggest that financial barriers have greater impact on access to dental services among elderlies living in rural areas [56]. In addition, in line with the literature, it was found in our study that presence of chronic diseases and poor general health are associated with less use of dental care [41].

Only small percentage of the participants reported forgoing dental care due to cost or unavailability of dental services. After adding welfare state regimes to the analysis, the main SES predictors (income level and unemployed status) remained significant. Participants living in the Eastern and Southern European countries were more likely to experience inadequate access to dental services, even after accounting for confounders. This is partly due to the design and financing of the health care system and partly due to the less favourable socioeconomic situation of the population [58].

The multiple barriers to access dental care services by elderly require specific actions [59]. One of the leading reasons, however, is the financial barrier, which requires interference in the insurance system and even a rethinking of the separation of dental care versus other medical care. Several policy options have been proposed to reduce the financial barriers to dental care. Potential fiscal policy solutions fall into two broad categories: extending dental insurance coverage and reforming population coverage to provide increased financial protection [60].

Since medical service utilisation is associated with increased probability of dental attendance [61], integration of dental with medical services could be a possible way to increase accessibility and utilisation of dental care [57]. In older age groups, there is a need for closer integration of dental care and medical care; dental care being typically provided by dentists today separately from the (primary) care team. Furthermore, health promotion and social policies should start at an early stage of life, as well as more policy actions should enhance and promote the need to seek oral care regularly and early, ensuring adequate accessibility to older age groups [62]. Although little is known about the extent to which interventions can change oral health behavior [63], the promotion of a healthy lifestyle is therefore seen as the gateway to healthy ageing [64].

### Strength and limitations

To the best of our knowledge, this is the first in-depth study to investigate longitudinally the use of dental and medical care among the elderly population in European countries, taking into account socio-economic factors and welfare state characteristics when comparing the use of different services. Data were used from nationally representative SHARE study. A further strength of our study is the large sample size. In addition, our findings are based on longitudinal data (7 years follow-up) allowing the causal inference. Given the longitudinal study design, we could measure how variations in socio-economic and health-related circumstances affect dental attendance. Such high-quality results can provide the basis for evidence-based health policy decisions to refocus the organisation and financing of dental care.

Our study has limitations due to data availability, restricting our analysis to only the data available in the four waves and not being able to include lifestyle factors such as diet, smoking and physical activity. Further limitation is that data on current oral health status and number of teeth of respondents were not incorporated into the models due to their unavailability in all waves, however, we used these variables in the sensitivity analyses from all the available years.

Our analysis was based on self-reported information on SES and health status which may lead to potential bias. Self-reported responses may be affected by recall bias, as elderlies were asked about past visits of medical and dental care. Furthermore, medical care provides a broader range of services, which may also influence the higher use of medical care compared to dental care, however, we adjusted for the number of chronic diseases and self-reported health status in the multivariate analysis. Another limitation may be that selective attrition may bias our results, which is common in longitudinal studies focusing on older adults [65]. Since attrition in older age groups is more related to decreased health status [66], the participants in our longitudinal sample may have better general and oral health than the general older population. Although, there were no demographic differences between participants who remained in the study and those who were not available across all waves, with the only exception for education—those who were not eligible to the longitudinal setting were less educated. Due to this dropout, our results might underestimate the strength of association between SES and use of services. Further research is needed to include a wider range of explanatory factors, including indicators of oral health and frailty.

## Conclusion

This longitudinal study found that inequalities in utilisation of dental care exist between welfare state regimes and are broader among social groups compared to other forms of care. The elderly population could benefit from adopting policies aiming to reduce the barriers to access to dental care and promote the need to seek regular and early dental care, especially in Southern and Eastern European countries. Given the relationship between chronic diseases and oral health, it would be worthwhile to explore the changes in quality of life and health care costs with regard to access to and utilisation of dental care in the elderly.

## Supporting information

S1 Fig Trend for dental care attendance during the preceding 12 months of interviews by baseline socioeconomic characteristics of respondents (income, education, employment status, and residential area). (TIF)

S2 Fig Trend for medical care attendance during the preceding 12 months of interviews by baseline socioeconomic characteristics of respondents (income, education, employment status, and residential area). (TIF)

S3 Fig Trend for hospitalization during the preceding 12 months of interviews by baseline socioeconomic characteristics of respondents (income, education, employment status, and residential area). (TIF)

S1 Table Relative (RII) and absolute (SII) inequalities related to dental care attendance in wave 5. (DOCX)

S2 Table Relative (RII) and absolute (SII) inequalities related to dental care, healthcare attendance and hospitalization by welfare state. (DOCX)

S3 Table Multilevel logistic regression model for determinants of dental care attendance, with imputed self-reported oral health and number of teeth to the models. (DOCX)

S4 Table Multilevel logistic regression analysis for forgoing dental care due to cost and unavailability in wave 8. (DOCX)
