# Supplementary figures and images for: Trends in dental care utilisation among the elderly using longitudinal data from 14 European countries: A multilevel analysis
Source: PLoS One. 2023 Jun 9;18(6):e0286192. doi: 10.1371/journal.pone.0286192 (PMC10256212; doi:10.1371/journal.pone.0286192)

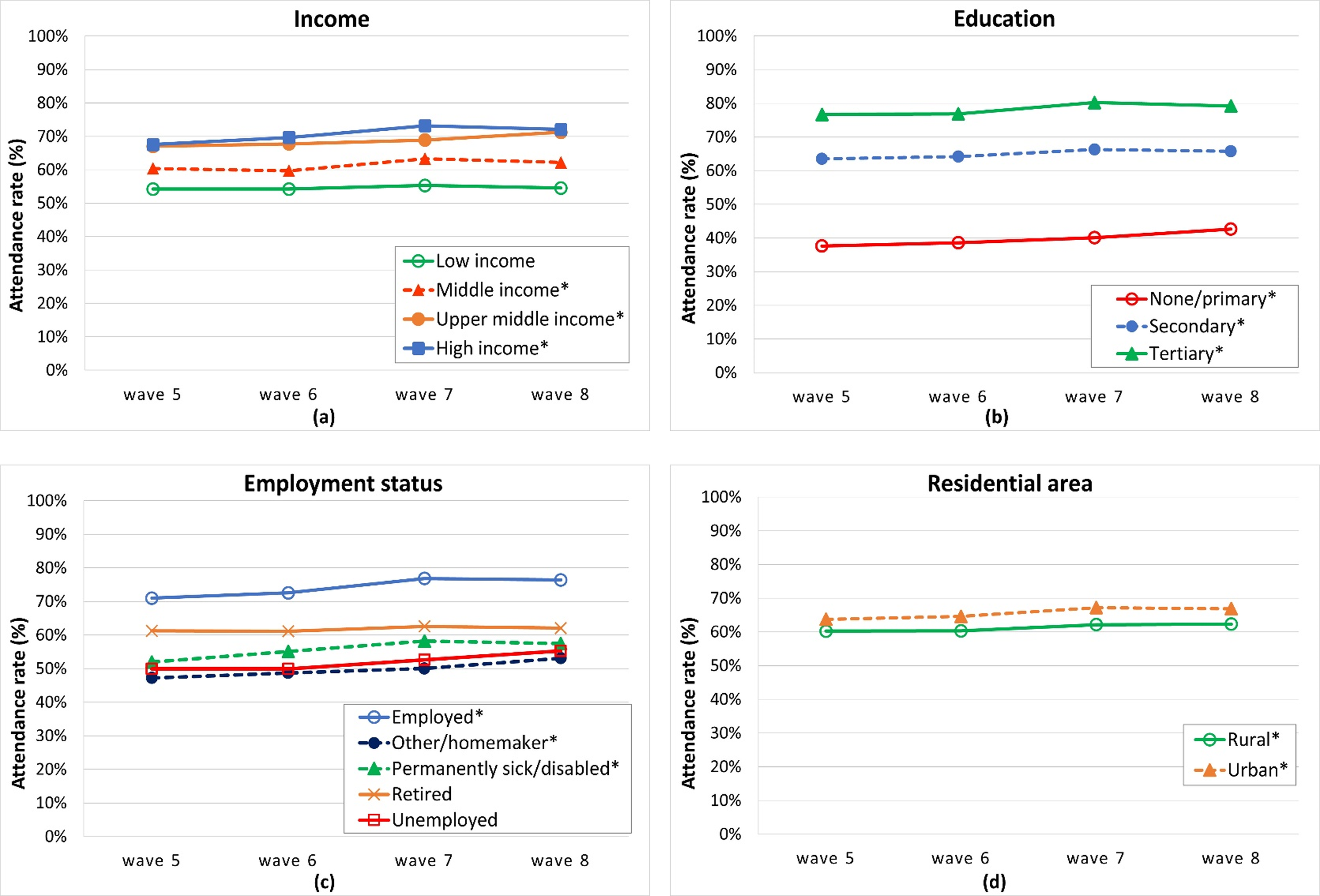

Supplement: S1 Fig — (TIF) [file pone.0286192.s001.tif]

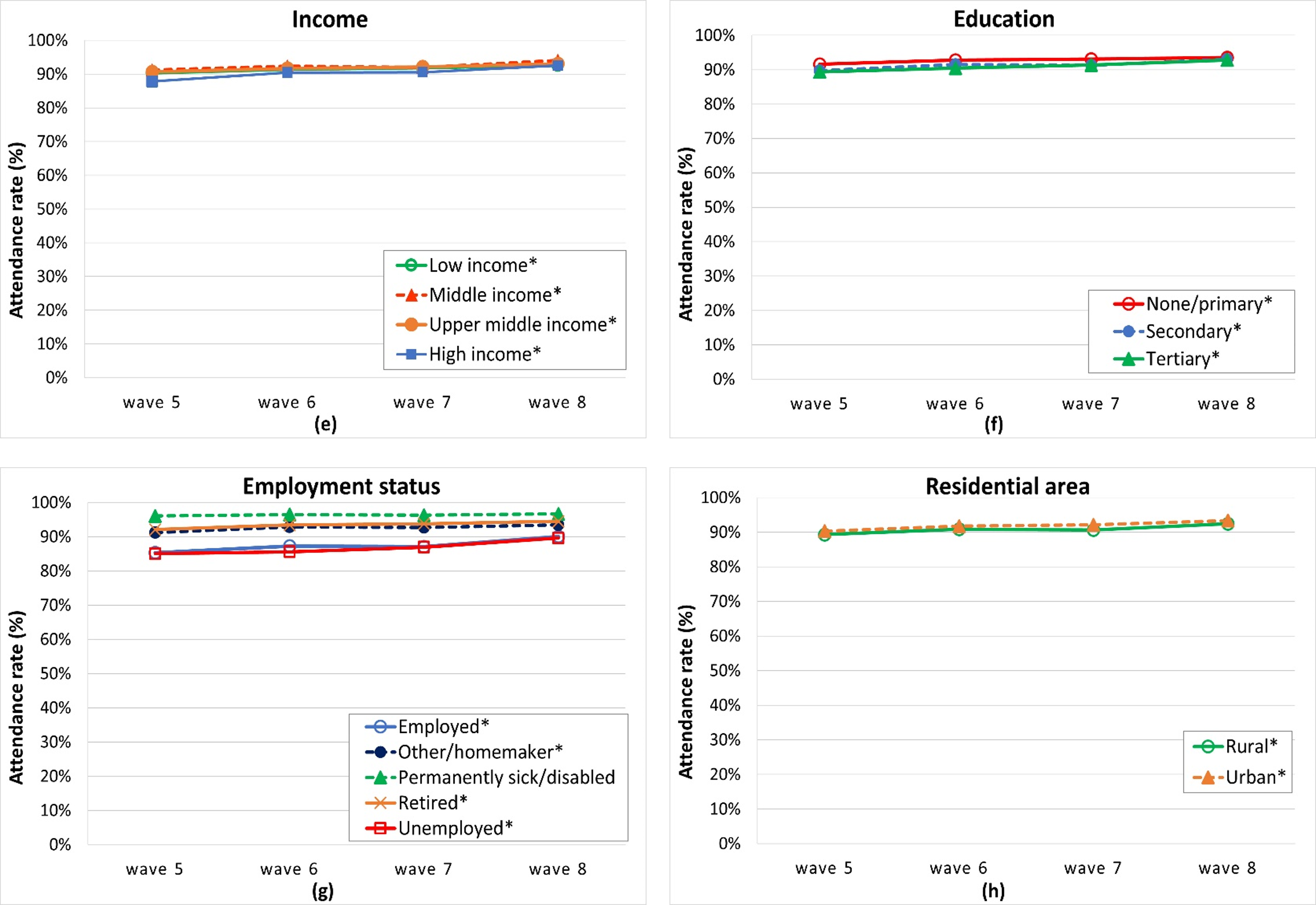

Supplement: S2 Fig — (TIF) [file pone.0286192.s002.tif]

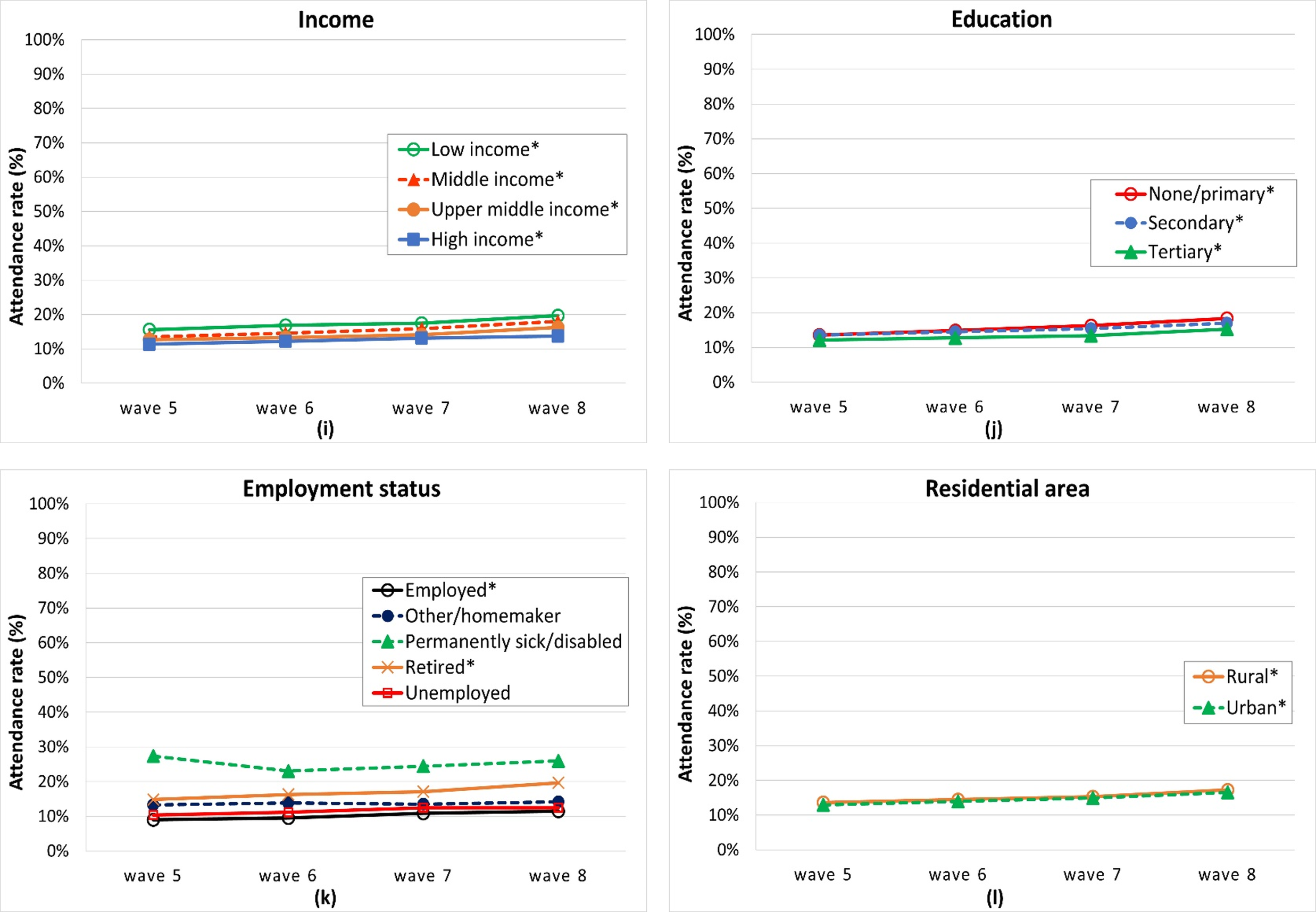

Supplement: S3 Fig — (TIF) [file pone.0286192.s003.tif]
