# Supplementary material for: Trends in dental care utilisation among the elderly using longitudinal data from 14 European countries: A multilevel analysis
Source: PLoS One. 2023 Jun 9;18(6):e0286192. doi: 10.1371/journal.pone.0286192 (PMC10256212; doi:10.1371/journal.pone.0286192)
Supplement: S1 Table — (DOCX) [file pone.0286192.s004.docx]

**Sensitivity analysis**

**S1 Table.** **Relative (RII) and absolute (SII) inequalities related to dental care attendance in wave 5**

|  | **RII (95% CI)** | **SII (95% CI)** |
| --- | --- | --- |
|  | wave5 | wave5 |
| Education | 1.63 (1.57-1.7)** | 0.351 (0.324-0.378)** |
|  |  |  |
| Income | 1.08 (1.04-1.12)** | 0.066 (0.041-0.09)** |

RII, Relative Index of Inequality, SII Slope Index of Inequality

All models were adjusted for age, gender, self-perceived health, number of chronic diseases, medication use, activity limitation, country, pain in teeth/mouth and number of teeth

*p<0.05, **p<0.001

#p-value for trend
