# Supplementary material for: Trends in dental care utilisation among the elderly using longitudinal data from 14 European countries: A multilevel analysis
Source: PLoS One. 2023 Jun 9;18(6):e0286192. doi: 10.1371/journal.pone.0286192 (PMC10256212; doi:10.1371/journal.pone.0286192)
Supplement: S2 Table — (DOCX) [file pone.0286192.s005.docx]

**S2 Table.** **Relative (RII) and absolute (SII) inequalities related to dental care, healthcare attendance and hospitalization by welfare state.**

|  | **RII (95% CI)** | | | | |
| --- | --- | --- | --- | --- | --- |
|  | **wave5** | **wave6** | **wave7** | **wave8** | **p-value#** |
| **Education** |  |  |  |  |  |
| Dental care |  |  |  |  |  |
| Scandinavian | 1.13 (1.07-1.19)** | 1.13 (1.07-1.19)** | 1.15 (1.09-1.21)** | 1.18 (1.12-1.25)** | 0.192 |
| Bismarckian | 1.59 (1.51-1.67)** | 1.53 (1.46-1.62)** | 1.52 (1.45-1.59)** | 1.49 (1.42-1.56)** | 0.142 |
| Southern | 2.34 (1.84-2.96)** | 2.49 (1.98-3.13)** | 2.56 (2.06-3.17)** | 1.96 (1.62-2.37)** | 0.185 |
| Eastern European | 1.54 (1.37-1.72)** | 1.46 (1.31-1.63)** | 1.63 (1.47-1.8)** | 1.57 (1.42-1.75)** | 0.315 |
| Medical care |  |  |  |  |  |
| Scandinavian | 1.06 (1.01-1.12)* | 1.03 (0.98-1.08) | 1.1 (1.05-1.16)** | 1.06 (1.02-1.11)* | 0.306 |
| Bismarckian | 1.05 (1.03-1.07)** | 1.02 (0.99-1.04) | 1.03 (1.01-1.06)* | 1.05 (1.03-1.07)** | 0.394 |
| Southern | 1.04 (0.99-1.09) | 1.03 (0.98-1.08) | 0.99 (0.94-1.04) | 1.03 (0.99-1.07) | 0.417 |
| Eastern European | 1.02 (0.98-1.06) | 1.03 (0.99-1.07) | 1.03 (0.99-1.06) | 1.05 (1.02-1.09)* | 0.109 |
| Hospitalization |  |  |  |  |  |
| Scandinavian | 0.96 (0.66-1.41) | 0.97 (0.68-1.37) | 0.85 (0.6-1.21) | 0.82 (0.6-1.11) | 0.323 |
| Bismarckian | 1.28 (1.06-1.55)* | 1.22 (1.02-1.46)* | 0.96 (0.81-1.15) | 1.12 (0.96-1.32) | 0.321 |
| Southern | 1.49 (0.94-2.35) | 0.82 (0.51-1.32) | 1.07 (0.69-1.65) | 0.99 (0.66-1.49) | 0.389 |
| Eastern European | 1.21 (0.87-1.66) | 0.88 (0.64-1.21) | 0.92 (0.68-1.23) | 1.02 (0.78-1.35) | 0.566 |
|  |  |  |  |  |  |
| **Income** |  |  |  |  |  |
| Dental care |  |  |  |  |  |
| Scandinavian | 1.11 (1.05-1.17)** | 1.18 (1.11-1.25)** | 1.17 (1.11-1.24)** | 1.22 (1.15-1.29)** | 0.146 |
| Bismarckian | 1.2 (1.14-1.25)** | 1.29 (1.23-1.35)** | 1.3 (1.24-1.36)** | 1.35 (1.29-1.41)** | 0.002 |
| Southern | 1.62 (1.31-2.02)** | 2.2 (1.76-2.74)** | 3.02 (2.44-3.72)** | 2.58 (2.15-3.11)** | 0.002 |
| Eastern European | 1.38 (1.25-1.52)** | 1.26 (1.14-1.38)** | 1.42 (1.29-1.56)** | 1.24 (1.13-1.36)** | 0.823 |
| Medical care |  |  |  |  |  |
| Scandinavian | 1.06 (0.99-1.12) | 1.06 (0.99-1.11)* | 1.05 (0.99-1.11) | 1.03 (0.99-1.08) | 0.681 |
| Bismarckian | 1.04 (1.02-1.06)* | 1.05 (1.03-1.07)** | 1.05 (1.03-1.07)** | 1.05 (1.03-1.07)** | 0.112 |
| Southern | 1.04 (1-1.09) | 1.03 (0.98-1.07) | 1.07 (1.02-1.11)* | 0.99 (0.96-1.03) | 0.942 |
| Eastern European | 1.06 (1.03-1.1)** | 1.03 (1.00-1.06) | 1.02 (0.99-1.05) | 1.04 (1.01-1.06)* | 0.396 |
| Hospitalization |  |  |  |  |  |
| Scandinavian | 1.07 (0.73-1.58) | 1.18 (0.81-1.7) | 0.73 (0.51-1.03) | 0.58 (0.42-0.81)* | 0.002 |
| Bismarckian | 1.03 (0.86-1.23) | 1.11 (0.94-1.31) | 0.96 (0.81-1.13) | 1.1 (0.95-1.28) | 0.886 |
| Southern | 1.12 (0.75-1.65) | 1.36 (0.92-2) | 1.29 (0.88-1.88) | 0.83 (0.58-1.19) | 0.188 |
| Eastern European | 1.31 (1.02-1.7)* | 1.13 (0.88-1.46) | 1.19 (0.92-1.53) | 1.07 (0.84-1.35) | 0.322 |
|  |  |  |  |  |  |
|  | **SII (95% CI)** | | | | |
|  | **wave5** | **wave6** | **wave7** | **wave8** | **p-value#** |
| **Education** |  |  |  |  |  |
| Dental care |  |  |  |  |  |
| Scandinavian | 0.14 (0.09-0.19)** | 0.13 (0.07-0.18)** | 0.12 (0.07-0.17)** | 0.12 (0.07-0.17)** | 0.463 |
| Bismarckian | 0.35 (0.31-0.39)** | 0.29 (0.26-0.34)** | 0.29 (0.26-0.34)** | 0.29 (0.26-0.34)** | 0.197 |
| Southern | 0.26 (0.18-0.34)** | 0.32 (0.24-0.4)** | 0.35 (0.27-0.43)** | 0.35 (0.27-0.43)** | 0.966 |
| Eastern European | 0.28 (0.21-0.35)** | 0.23 (0.16-0.3)** | 0.32 (0.25-0.39)** | 0.32 (0.25-0.39)** | 0.597 |
| Medical care |  |  |  |  |  |
| Scandinavian | 0.03 (-0.02-0.09) | 0.02 (-0.03-0.07) | 0.11 (0.06-0.17)** | 0.06 (0.01-0.11)* | 0.111 |
| Bismarckian | 0.04 (0.02-0.07)* | 0.01 (-0.02-0.03) | 0.04 (0.01-0.06)* | 0.04 (0.02-0.07)* | 0.32 |
| Southern | 0.03 (-0.02-0.09) | 0.03 (-0.02-0.08) | -0.02 (-0.07-0.03) | 0.01 (-0.04-0.06) | 0.396 |
| Eastern European | 0.01 (-0.04-0.06) | 0.03 (-0.02-0.08) | 0.03 (-0.02-0.08) | 0.08 (0.03-0.13)* | 0.032 |
| Hospitalization |  |  |  |  |  |
| Scandinavian | -0.02 (-0.07-0.02) | -0.02 (-0.07-0.03) | -0.02 (-0.07-0.03) | -0.05 (-0.1-0) | 0.256 |
| Bismarckian | 0.01 (-0.02-0.04) | 0.02 (-0.02-0.05) | -0.02 (-0.05-0.02) | -0.02 (-0.06-0.01) | 0.097 |
| Southern | 0.03 (-0.01-0.08) | -0.01 (-0.05-0.04) | 0.002 (-0.05-0.06) | -0.03 (-0.08-0.02) | 0.046 |
| Eastern European | 0.004 (-0.04-0.05) | -0.004 (-0.05-0.04) | -0.01 (-0.06-0.04) | 0.02 (-0.03-0.07) | 0.951 |
|  |  |  |  |  |  |
| **Income** |  |  |  |  |  |
| Dental care |  |  |  |  |  |
| Scandinavian | 0.12 (0.07-0.17)** | 0.16 (0.11-0.21)** | 0.13 (0.08-0.18)** | 0.17 (0.12-0.23)** | 0.417 |
| Bismarckian | 0.14 (0.11-0.18)** | 0.2 (0.16-0.23)** | 0.2 (0.16-0.23)** | 0.23 (0.19-0.26)** | 0.003 |
| Southern | 0.16 (0.09-0.23)** | 0.24 (0.17-0.31)** | 0.34 (0.27-0.42)** | 0.34 (0.26-0.42)** | <0.001 |
| Eastern European | 0.19 (0.13-0.25)** | 0.13 (0.07-0.19)** | 0.23 (0.17-0.29)** | 0.15 (0.09-0.21)** | 0.862 |
| Medical care |  |  |  |  |  |
| Scandinavian | 0.04 (-0.01-0.09) | 0.04 (-0.01-0.09) | 0.05 (-0.004-0.1) | 0.02 (-0.03-0.07) | 0.770 |
| Bismarckian | 0.03 (0.01-0.05)* | 0.04 (0.02-0.06)** | 0.05 (0.03-0.07)** | 0.05 (0.03-0.08)** | 0.035 |
| Southern | 0.04 (-0.01-0.08) | 0.04 (-0.003-0.09) | 0.05 (0.01-0.1)* | 0 (-0.05-0.04) | 0.608 |
| Eastern European | 0.06 (0.02-0.1)* | 0.04 (0.002-0.07)* | 0.03 (-0.01-0.07) | 0.05 (0.02-0.09)* | 0.668 |
| Hospitalization |  |  |  |  |  |
| Scandinavian | -0.01 (-0.05-0.03) | 0 (-0.05-0.05) | -0.04 (-0.09-0.01) | -0.09 (-0.14--0.05)** | 0.004 |
| Bismarckian | -0.01 (-0.04-0.01) | 0.004 (-0.02-0.03) | -0.02 (-0.05-0.01) | -0.002 (-0.03-0.03) | 0.876 |
| Southern | 0.01 (-0.03-0.05) | 0.03 (-0.01-0.08) | 0.03 (-0.01-0.08) | -0.001 (-0.05-0.05) | 0.532 |
| Eastern European | 0.02 (-0.02-0.06) | 0.02 (-0.02-0.06) | 0.03 (-0.02-0.07) | 0.01 (-0.03-0.06) | 0.752 |

RII, Relative Index of Inequality, SII Slope Index of Inequality

All models were adjusted for age, gender, self-perceived health, number of chronic diseases, medication use, activity limitation and country.

*p<0.05, **p<0.001

#p-value for trend
