# Supplementary material for: Trends in dental care utilisation among the elderly using longitudinal data from 14 European countries: A multilevel analysis
Source: PLoS One. 2023 Jun 9;18(6):e0286192. doi: 10.1371/journal.pone.0286192 (PMC10256212; doi:10.1371/journal.pone.0286192)
Supplement: S3 Table — (DOCX) [file pone.0286192.s006.docx]

**Sensitivity analysis**

**S3 Table. Multilevel logistic regression model for determinants of dental care attendance, with imputed self-reported oral health and number of teeth to the models**

|  | Model 1  (N=20,803) | Model 2  (N=20,423) |
| --- | --- | --- |
| **Age, years (Reference: 50-54)** |  |  |
| 55-59 | 1.14 (1.04-1.25)* | 1.14 (1.01-1.3)* |
| 60-64 | 1.13 (1.03-1.24)* | 1.09 (0.95-1.25) |
| 65-69 | 1.06 (0.96-1.17) | 1.15 (0.99-1.35) |
| 70-74 | 0.99 (0.89-1.1) | 1.12 (0.95-1.33) |
| >75 | 0.69 (0.63-0.77)** | 0.95 (0.81-1.13) |
| **Gender (Reference: Female)** |  |  |
| Male | 0.71 (0.68-0.73)** | 0.77 (0.72-0.82)** |
| **Education (Reference: None/primary)** |  |  |
| Secondary | 1.64 (1.56-1.72)** | 1.57 (1.42-1.74)** |
| Tertiary | 2.48 (2.34-2.63)** | 2.14 (1.9-2.41)** |
| **Household income (Reference: Low income)** |  |  |
| Middle income | 1.32 (1.26-1.38)** | 1.16 (1.06-1.28)* |
| Upper middle income | 1.58 (1.51-1.66)** | 1.4 (1.27-1.54)** |
| High income | 1.8 (1.71-1.89)** | 1.32 (1.2-1.47)** |
| **Residential area Reference: Rural)** |  |  |
| Urban | 1.15 (1.11-1.19)** | 1.13 (1.05-1.21)* |
| **Employment status (Reference: Employed)** |  |  |
| Other/homemaker | 0.84 (0.77-0.91)** | 0.93 (0.8-1.08) |
| Permanently sick | 0.81 (0.72-0.91)** | 0.82 (0.66-1.01) |
| Retired | 0.97 (0.91-1.03) | 1.09 (0.97-1.22) |
| Unemployed | 0.7 (0.61-0.79)** | 0.76 (0.61-0.93)* |
| **Number of chronic diseases (Reference: 0)** |  |  |
| 1 | 1.08 (1.03-1.14)** | 1.15 (1.04-1.28)* |
| 2 or more | 1.17 (1.1-1.24)** | 1.39 (1.24-1.56)** |
| **Activity limitations (Reference: No)** | 0.97 (0.93-1.01) | 0.97 (0.9-1.04) |
| **Medication use (Reference: No)** | 1.11 (1.06-1.17)** | 1.1 (0.99-1.21) |
| **Self-perceived health (Reference: Excellent)** |  |  |
| Very good | 0.99 (0.91-1.07) | 0.85 (0.74-0.98)* |
| Good | 0.85 (0.79-0.92)** | 0.82 (0.71-0.94)* |
| Fair | 0.72 (0.66-0.78)** | 0.67 (0.58-0.79)** |
| Poor | 0.54 (0.49-0.6)** | 0.55 (0.45-0.66)** |
| **Welfare system (Reference: Scandinavian)** |  |  |
| Bismarckian | 0.49 (0.24-1.01) | 0.59 (0.29-1.2) |
| Southern | 0.1 (0.04-0.25)** | 0.1 (0.04-0.25)** |
| Transitional—East European | 0.18 (0.08-0.4)** | 0.26 (0.12-0.58)* |
| **Pain in teeth/mouth** | 2.26 (1.91-2.68)** | 2.86 (1.95-4.22)** |
| **Number of teeth** | - | 1.05 (1.05-1.06)** |
| **Wave (Reference: Wave 5)** |  |  |
| Wave 6 | 1.05 (1.01-1.1)* | - |
| Wave 7 | 1.24 (1.18-1.3)** | - |
| Wave 8 | 1.28 (1.22-1.34)** | - |
| Intercept | 2.75 (1.44-5.24)** | 0.89 (0.46-1.72) |

In Model 1, multiple imputation was used regarding missing data in wave 7, with pain in teeth/mouth variable (N= 14,389) being imputed.

In Model 2, the analysis was performed in the baseline year (in wave 5), and pain in teeth/mouth and number of teeth (available only in wave 5) variables were added to the model.
