# Supplementary material for: Trends in dental care utilisation among the elderly using longitudinal data from 14 European countries: A multilevel analysis
Source: PLoS One. 2023 Jun 9;18(6):e0286192. doi: 10.1371/journal.pone.0286192 (PMC10256212; doi:10.1371/journal.pone.0286192)
Supplement: S4 Table — (DOCX) [file pone.0286192.s007.docx]

**S4 Table.** **Multilevel logistic regression analysis for forgoing dental care due to cost and unavailability in wave 8**

|  | **Forgoing dental care due to cost** | | **Forgoing dental care due to unavailability** | |
| --- | --- | --- | --- | --- |
|  | **Model 1** | **Model 2** | **Model 1** | **Model 2** |
|  | OR (95% CI) | OR (95% CI) | OR (95% CI) | OR (95% CI) |
| **Age, years (Reference: 50-54)** |  |  |  |  |
| 60-64 | 0.55 (0.4-0.76)** | 0.55 (0.4-0.76)** | 0.67 (0.39-1.17) | 0.67 (0.38-1.16) |
| 65-69 | 0.4 (0.27-0.57)** | 0.4 (0.27-0.57)** | 0.43 (0.23-0.81)* | 0.42 (0.23-0.79)* |
| 70-74 | 0.35 (0.24-0.51)** | 0.35 (0.24-0.51)** | 0.37 (0.19-0.7)* | 0.36 (0.19-0.69)* |
| >75 | 0.19 (0.13-0.27)** | 0.19 (0.13-0.27)** | 0.24 (0.13-0.45)** | 0.23 (0.12-0.44)** |
| **Gender (Reference: Female)** |  |  |  |  |
| Male | 1.02 (0.86-1.21) | 1.02 (0.86-1.21) | 1.25 (0.95-1.66) | 1.26 (0.95-1.66) |
| **Education (Reference: None/primary)** |  |  |  |  |
| Secondary | 0.96 (0.76-1.23) | 0.97 (0.76-1.24) | 0.95 (0.65-1.39) | 0.95 (0.65-1.39) |
| Tertiary | 1.02 (0.76-1.37) | 1.03 (0.77-1.39) | 0.91 (0.56-1.49) | 0.93 (0.57-1.51) |
| **Household income (Reference: Low income)** |  |  |  |  |
| Middle income | 0.64 (0.52-0.8)** | 0.64 (0.52-0.8)** | 0.65 (0.46-0.91)** | 0.65 (0.46-0.91)* |
| Upper middle income | 0.53 (0.42-0.67)** | 0.53 (0.42-0.67)** | 0.42 (0.28-0.62)* | 0.41 (0.28-0.62)* |
| High income | 0.35 (0.27-0.45)** | 0.35 (0.27-0.45)** | 0.46 (0.31-0.68)** | 0.46 (0.31-0.67)** |
| **Residential area Reference: Rural)** |  |  |  |  |
| Urban | 1.17 (0.98-1.39) | 1.16 (0.97-1.38) | 1.2 (0.9-1.59) | 1.19 (0.89-1.58) |
| **Employment status (Reference: Employed)** |  |  |  |  |
| Other/homemaker | 1.75 (1.18-2.59)* | 1.73 (1.17-2.57)* | 1.33 (0.67-2.64) | 1.31 (0.66-2.6) |
| Permanently sick | 1.69 (1.08-2.65)* | 1.69 (1.08-2.65)* | 0.93 (0.39-2.23) | 0.92 (0.38-2.22) |
| Retired | 1.29 (0.94-1.77) | 1.29 (0.94-1.77) | 1.3 (0.77-2.19) | 1.31 (0.78-2.22) |
| Unemployed | 2.36 (1.38-4.03)* | 2.34 (1.37-4)* | 2.42 (1.05-5.6)* | 2.36 (1.02-5.47)* |
| **Number of chronic diseases (Reference: 0)** |  |  |  |  |
| 1 | 1.01 (0.74-1.39) | 1.01 (0.74-1.39) | 0.83 (0.48-1.41) | 0.83 (0.49-1.43) |
| 2 or more | 1.46 (1.05-2.02)* | 1.46 (1.06-2.02)* | 1.13 (0.66-1.94) | 1.15 (0.67-1.96) |
| **Activity limitations (Reference: No)** | 1.22 (1-1.5) | 1.23 (1-1.51) | 1.48 (1.05-2.08)* | 1.48 (1.06-2.09)* |
| **Medication use (Reference: No)** | 0.83 (0.62-1.09) | 0.82 (0.62-1.09) | 0.91 (0.56-1.48) | 0.9 (0.55-1.47) |
| **Self-perceived health (Reference: Excellent)** |  |  |  |  |
| Very good | 1.21 (0.75-1.94) | 1.2 (0.75-1.94) | 1.67 (0.57-4.9) | 1.65 (0.56-4.84) |
| Good | 1.21 (0.77-1.92) | 1.21 (0.76-1.91) | 2.02 (0.71-5.68) | 1.96 (0.7-5.53) |
| Fair | 1.57 (0.97-2.55) | 1.56 (0.96-2.53) | 2.86 (0.99-8.3) | 2.76 (0.95-7.98) |
| Poor | 1.98 (1.17-3.36)* | 1.96 (1.16-3.33)* | 3.45 (1.13-10.51)* | 3.29 (1.08-10.03)* |
| **Welfare system (Reference: Scandinavian)** |  |  |  |  |
| Bismarckian |  | 0.7 (0.29-1.68) |  | 1.29 (0.47-3.53)* |
| Southern |  | 1.54 (0.52-4.56) |  | 3.71 (1.14-12.04)* |
| Transitional—East European |  | 1.34 (0.5-3.61) |  | 4 (1.36-11.72)* |
| Intercept | 0.05 (0.03-0.1)** | 0.05 (0.02-0.14)** | 0.01 (0-0.03)** | 0 (0-0.02)** |
| ICC (%) | 10.77% | 7.81% | 15.39% | 7.05% |
| AIC | 5333.046 | 5334.87 | 2408.099 | 2404.818 |

Total participants = 20,773, N groups = 14. *p<0.05, **p<0.001

OR, Odds Ratio, CI, Confidence interval, AIC, Akaike Information Criterion, ICC, Intraclass Correlation coefficient.
